# A Love Rooted Deep in the Appalachian Mountains: One Part of the Legacy of Doug Scutchfield

**DOI:** 10.13023/jah.0402.01

**Published:** 2022-07-01

**Authors:** Charlotte S. Seidman

**Affiliations:** Seidman Writing, charlotte.seidman@uky.edu

**Keywords:** legacy, public health, preventive medicine, obituary

## Abstract

F. Douglas Scutchfield, MD, died on Monday, May 23, 2022 in Lexington KY.

I have known and worked with Doug for over 40 years and share my personal insights into how he created the Journal of Appalachian Health, one of his last great career adventures and a lasting legacy to his deep investment in the health and vitality of Appalachia and its people.

I lost a great friend on Monday, May 23rd, and public health lost a great champion. I met Doug Scutchfield in 1981 when I was a student entering the first class of the new Graduate School of Public Health at San Diego State University. I didn’t know at the time that ours would be a lifelong friendship on both a personal and a professional level.

He was one of the most important forces in my professional life, as he was to myriad students in both San Diego and Kentucky. We spent almost 20 years working as the editorial team—along with Editor-in-Chief Kevin Patrick—of the *American Journal of Preventive Medicine*, and when he contacted me 5 years ago to see if I’d be interested in starting a new journal with him, I jumped at the chance.

We were both products of the Appalachian Mountains—he was born in Kentucky; I was born in central Pennsylvania—and wanted our last career adventure to be in service of the health of the people in those mountain communities. We worked for over a year developing the proposal and securing funding for this new journal.

Doug spent hours thinking through how best to set a firm foundation for the journal, choosing the finest representatives from the universities in the 13-state Appalachian region, and also focusing on the importance of those outside the typical academic settings, incorporating people from a wide variety of interests: the arts, religion, hospitals, foundations, wellness centers, community organizations, rural health, community and economic development, social theory, racial justice, nonprofits, and advocates for the health of Appalachia.

He pulled together an outstanding editorial board and added an equally impressive advisory board. Under the guidance of these leaders, he created the underpinning for what would become the *Journal of Appalachian Health*.

His work paid off in ways that we could not imagine at the time. Now in our fourth year of publication, we recently applied for and received inclusion in PubMed Central. It was Doug’s foresight that allowed for our success on our first application. He had given us the excellence we needed to establish the journal as one worthy to be included in the NIH/NLM database.

For a lifetime of friendship, mentorship, and just plain old common sense, I thank him. As he always said: “When you’re up to your ass in alligators, it’s hard to remember that your job is to drain the swamp.”

For all those swamps you drained, Doug, the public health world applauds you.

## Supplementary Information



