# Supplementary figures and images for: A Love Rooted Deep in the Appalachian Mountains: One Part of the Legacy of Doug Scutchfield
Source: J Appalach Health. 2022 Jul 1;4(2):1–3. doi: 10.13023/jah.0402.01 (PMC10629877; doi:10.13023/jah.0402.01)

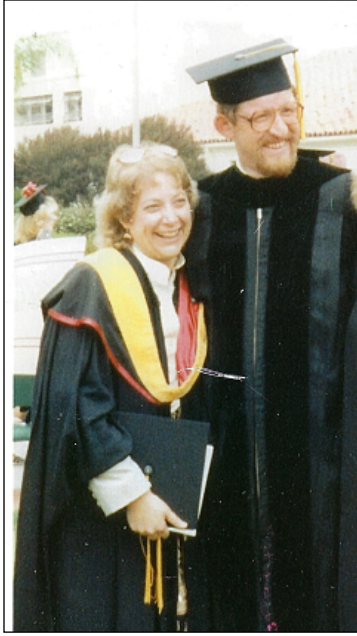

Charlotte Seidman and Doug Scutchfield  
San Diego State University  
First GSPH Graduation, May 1982

Supplement: Supplementary file 1 [file 4.2.1_Seidman_AdditionalFile.pdf]
